# Clinical and MRI manifestations of nitrous oxide induced vitamin B12 deficiency: A case report

**Published:** 2013

**Authors:** Anahid Safari, Farhad Emadi, Elham Jamali, Afshin Borhani-Haghighi

**Affiliations:** 1 Department of Pharmacology, School of Medicine, Azad University, Kazerun Branch, Shiraz, Iran; 2 Health Policy Research Center, Shiraz University of Medical Sciences, Shiraz, Iran; 3 Research Center for Traditional Medicine and History of Medicine AND Department of Neurology, Shiraz University of Medical Sciences, Shiraz, Iran

**Keywords:** Ataxia, Spinal Cord, General Anesthesia, Nitrous Oxide, Vitamin B12 Deficiency

## Abstract

A 50 year-old man was referred with history of acute ataxia and lower extremity paresthesia 10 days after general anesthesia with nitrous oxide. Cervical MRI showed long hypersignal lesion in posterior segment of the cord. Blood analysis revealed vitamin B12 deficiency. Nitrous oxide-induced myelopathy should be considered in patients who develop acute neurological manifestation after general anesthesia. It is recommended for physicians to think about symptoms and signs of B12 deficiency when evaluating patients in postoperative visits.

## Introduction

Nitrous oxide is an inhaled anesthetic drug which irreversibly oxidizes the cobalt ion of cobalamin (vitamin B12) from the (+) 1 to the (+) 3 valence state. Oxidation of the cobalt ion by nitrous oxide prevents methylcobalamin from acting as a coenzyme in the production of methionine and subsequently *S*-adenosylmethionine, which is necessary for methylation of myelin sheath phospholipids. The result is decreased myelin formation. In addition, the conversion of methylmalonyl to succinyl coenzyme is inhibited as a result of cobalamin oxidation.^1^

Accumulation of methylmalonate and propionate may provide abnormal substrates for fatty acid synthesis, and subsequently these abnormal fatty acids may be incorporated into the myelin sheath.^1, 2^ The end result of nitrous oxide toxicity appears to be subacute combined degeneration of the spinal cord, as described in classic vitamin B12 deficiency with classic picture of subacute combined degeneration of the dorsal (posterior) and lateral spinal columns.^3^ The related neuropathy is symmetrical and affects the legs more than the arms. It begins with paresthesia and ataxia associated with loss of vibration and position sense, and can progress to severe weakness, spasticity, clonus, paraplegia, and even fecal and urinary incontinence. Other neurologic abnormalities that can be seen include axonal degeneration of peripheral nerves, and central nervous system symptoms including memory loss, irritability, and dementia. Patients may present with Lhermitte's syndrome; a shock-like sensation that radiates to the feet during neck flexion.^4, 5^

## Case Report

A 50 year-old man was referred to the neurology clinic with 3 days history of ataxia.

**Figure 1:**
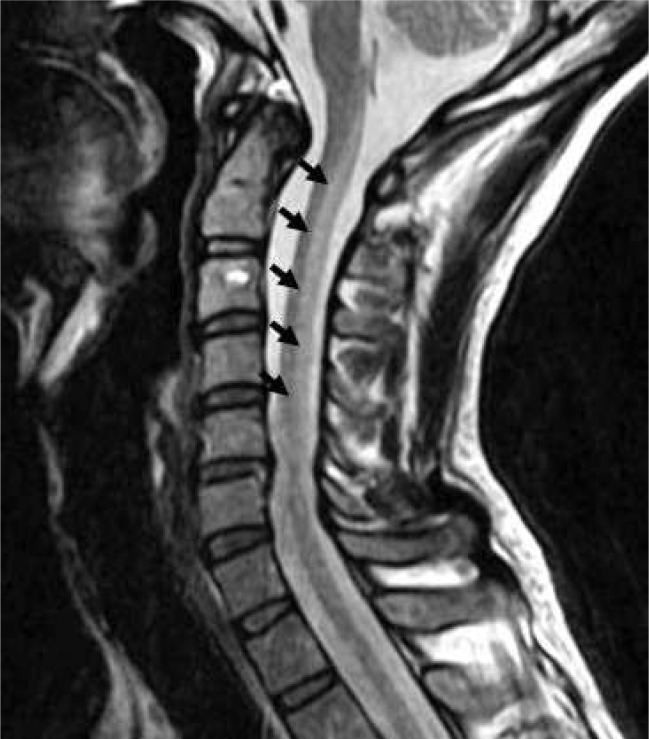
Sagittal T2 weighted MRI showing long (C2- C7) hypersignal lesion in posterior part of spinal cord (black arrows)

He had undergone tympanoplasty operation 2 weeks prior to referral. 2 days after the operation he developed anorexia, depressed mood, and burning sensation in lower extremities. A few days later he developed disequilibrium and ataxia, and then referred to our neurology clinic. The patient's background and history did not reveal pre-existing diabetes mellitus, alcohol consumption, vegetarian food preference, gastrointestinal symptoms, or previous neurological disorder. General examination did not show any abnormality. Neurological examination revealed normal mini-mental status, depressed mood, normal speech, and cranial nerves. Motor examination did not show any weakness, and deep tendon reflexes were normal. No pathologic reflex was present. Sensory examination was also normal for pinprick and temperature sensations. However, position and vibration sense were obviously impaired in both upper and lower extremities. Cerebellar examination was normal. The patient had marked sensory ataxia with positive Romberg's sign. Lhermitte's sign was also positive. Laboratory tests revealed WBC count: 6000, RBC count: 4.91 million/dl, Hb: 11.2, and MCV: 116. Furthermore, peripheral blood smear showed macro-ovalocytes and hypersegmented neutrophils, B12 level: 101, and homocysteine level: 209. Brain MRI was normal. Cervical MRI showed long T2 hypersignal lesion at posterior of cord from C2 to C7 (Figure 1). Treatment with parenteral B12 was started (supplementation of vitamin B12 1000 µg IM daily for a week, and then weekly for 4 weeks); and the patient was followed clinically and with lab data. The patient's neurological manifestations improved after 4 weeks of treatment with B12. We continued parenteral B12 monthly for this patient.

## Discussion

Cobalamin (vitamin B12) deficiency is the most common cause of megaloblastic anemia. Among patients aged > 65 years, between 10 to 15% have cobalamin deficiency.^5^ The causes of cobalamin deficiency are pernicious anemia, food-cobalamin malabsorption, other malabsorption syndromes, nutritional deficiency, and other gastrointestinal causes (e.g. Zollinger-Ellison syndrome, Crohn's disease, celiac disease, or chronic pancreatic insufficiency).^6^ MR imaging of the posterior column, and rarely lateral column, of the spinal cord that showed abnormally increased T2-signal hyperintensity in SCD have been documented.^7^ Differential diagnoses of abnormal signal lesions in the posterior columns of the spinal cord include infectious or postinfectious myelitis, peripheral neuropathy, lymphoma and other neoplasm, paraneoplastic myelopathy, cervical spondylosis, radiation myelitis, multiple sclerosis, sarcoidosis, arterial or venous ischemia, traumatic cord injury, arterial or venous ischemia, vascular malformations of the dura and spinal cord, syringomyelia, metabolic disease (including vitamin E deficiency), and acute transverse myelitis.^8^

Several investigators have reported the development of myelopathy 2 to 6 weeks after anesthesia with nitrous oxide for a variety of surgical procedures. All these patients were found to have underlying cobalamin deficiency that was unknown at the time of the procedure. Since approximately 14% of the population may have cobalamin deficiency, awareness of this process is critical.^1^ In patients with postsurgical myelopathy, the physician must consider the possibility that nitrous oxide toxicity has caused an unknown underlying B12 deficiency to become clinically overt. MR imaging may be helpful in suggesting the diagnosis.

## Conclusion

We recommend checking serum B12 level in patients who have undergone general anesthesia and then developed disequilibrium and ataxia. Moreover, it is recommended for physicians to think about symptoms and signs of B12 deficiency when evaluating patients in postoperative visits.
